# Supplementary material for: Stochastic modelling of delays and buffering in 5G-IoT ecosystems with programmable P4 switches based on BMAP
Source: PLoS One. 2025 Aug 29;20(8):e0330526. doi: 10.1371/journal.pone.0330526 (PMC12396653; doi:10.1371/journal.pone.0330526)
Supplement: Attachment — Submitted filename: Rebuttal letter.doc [file pone.0330526.s002.doc]

**Responses to the comments of Reviewer 1**

We sincerely thank Reviewer 1 for the detailed and constructive feedback. We appreciate the recognition of the originality, analytical rigor, and empirical foundation of our work. Your insightful comments have helped us identify areas for improvement in both the presentation and scope of our study. We have carefully considered each suggestion and have revised the manuscript and accompanying materials accordingly. Below, we provide a point-by-point response to each comment, detailing the changes made and clarifying the rationale behind our modeling choices.

**1. One major issue, in this reviewer opinion, lies in the use of 3D visualizations to present results. While intended to show parameter interactions, these plots often hinder clarity. In particular, Figs 6 and 8 can be replaced without loss, and Figs 7 and 9 don't bring any clarity to the results. Replacing them with 2D plots (e.g., heatmaps or line graphs) would improve the reader's ability to interpret trends and compare outcomes.**

We sincerely thank the reviewer for this insightful comment regarding the visual representation of results. We fully agree that clarity and interpretability should take precedence over graphical complexity. In response:

- Figures 6 and 8 have been entirely replaced by 2D line plots, which clearly show the dependency of delay on key parameters (e.g., processing rate and arrival rate) for fixed values of the other variable. This improves the readability and allows direct comparison across configurations.

- Figure 7, which originally illustrated the influence of buffer size under varying load conditions, has been replaced with two separate 2D projections (delay vs. arrival rate; delay vs. processing rate). This makes the trend and saturation effects more accessible and aligns better with the narrative of resource-bounded behavior.

- Figure 9, which depicted total delay under hybrid conditions, has been replaced by a heatmap, enabling clear identification of the stable and unstable zones in the (*q_i_*, *κ′*/*κ*) parameter space. This not only highlights the QoS sensitivity but also improves the reader’s ability to pinpoint transition thresholds.

All corresponding figure captions and in-text descriptions have been carefully revised to reflect these changes and ensure consistency with the theoretical analysis.

**2. Additionally, assumptions such as infinite buffers and simplified traffic sources may not accurately reflect real-world constraints. A broader evaluation with diverse traffic scenarios and comparison to other modeling techniques would help confirm the model's general applicability.**

We appreciate the reviewer’s thoughtful concern regarding model assumptions. The adopted abstraction of infinite buffer capacity and batch-based traffic generation is indeed a simplification. However, these choices were made intentionally, and they are well aligned with the focus and scope of the study, which targets theoretical modeling of processing delay dynamics in programmable P4-based switches under hybrid path architectures.

Specifically, the P4 pipeline model considered in this work reflects the behavior of deterministic, statically scheduled packet processing, where buffers are primarily internal per-stage queues with tight scheduling and bounded processing latency. The assumption of virtually non-blocking local buffers (approximated analytically as infinite) follows the approach taken in many prior analytical models of P4 and FPGA-accelerated architectures, where backpressure is minimized by design, and head-of-line blocking is rare under nominal load conditions.

As for the traffic model, batch arrivals are used to reflect scheduled telemetry bursts or batched packet exports common in in-band network telemetry (INT), monitoring, and load reporting applications (typical P4 use cases). The simplification enables tractable analysis of processing delay trends and parameter sensitivity, which is the primary goal of the study.

At the same time, we fully agree with the reviewer that addressing these idealizations would significantly expand the generalizability of the proposed mathematical apparatus. To this end, we plan the following theoretical and applied extensions in future work:

- On the theoretical side, we aim to extend the current model to finite-buffer systems with loss probabilities and explicitly model buffer occupancy distributions. This includes incorporating M^X^/G/1/K, BMAP/G/1/K, or MAP/PH/1/K queuing models to preserve burstiness and state-dependence while accounting for realistic capacity constraints.

- We also plan to generalize the arrival process to include ON/OFF modulated sources, self-similar input, and correlated service dependencies, thereby moving toward a framework suitable for variable, unpredictable traffic environments.

- On the applied side, we will validate the extended models through emulated environments (e.g., P4 bmv2, NetFPGA, or Tofino simulation tools). We will also conduct packet-level simulations using realistic INT traffic traces and stress-testing under various congestion and scheduling policies.

- Finally, we will perform comparative evaluations against other modeling approaches (e.g., discrete-event simulators and ML-based predictors) to measure trade-offs between tractability, accuracy, and deployment readiness.

We believe these directions will allow us to preserve the strengths of the current analytical framework while broadening its relevance to practical 5G-IoT deployments.

**3. How were the main model parameters (like BMAP rates and service times) estimated from the real dataset? Did you use any fitting method?**

We thank the reviewer for highlighting the need to clarify the parameter estimation methodology. In response, we have significantly expanded Section 3 to provide a detailed account of how the key model parameters (such as the BMAP arrival matrices and service time distributions) were derived from real-world traffic data.

Specifically, we now describe how the bursty input process was modeled as a low-order Batch Markovian Arrival Process (BMAP), fitted to IoT telemetry traces from the Kaggle repository. The fitting procedure involved aggregating packet arrivals over time windows, computing empirical moments, and applying the method of moments to construct initial estimates of the BMAP generator matrices. These were then refined using a least-squares approach to align the model’s autocorrelation structure with that observed in the dataset.

For the service process, we employed a hybrid approach: exponential or Erlang distributions were fitted to forwarding delays via maximum likelihood, while truncated normal distributions were used for control-plane delays, estimated via expectation-maximization. The resulting models were validated against empirical cumulative distribution functions (ECDFs) and histograms.

These additions ensure that the modeling methodology is transparent and well grounded in data, and they address the reviewer’s concern about fitting techniques. We appreciate this helpful suggestion.

**4. What is the impact of assuming infinite buffers? Would the results still hold under more realistic buffer limits?**

We appreciate the reviewer’s question regarding the impact of assuming infinite buffers. This assumption was used as a deliberate analytical simplification aligned with prior P4- and FPGA-based queuing models, as explained in our response to Comment 2. However, to address the specific point raised here, we clarify its implications more precisely.

Assuming infinite buffers eliminates packet drops and backpressure-induced delays from the model, thereby yielding lower-bound delay estimates under nominal conditions. This means that while the predicted average delay trends remain valid and analytically tractable, the model does not capture congestion effects or queue overflow risks that can arise in highly loaded systems with finite capacity.

To address this limitation, we have outlined (see response to Comment 2) our plan to extend the model using BMAP/G/1/K and related queuing frameworks. These models will enable us to compute loss probabilities, tail delays, and queue saturation thresholds, thus allowing us to assess how much the infinite-buffer approximation diverges from realistic deployments under varying load and burst conditions. We also intend to conduct stress-testing of these extensions in emulated programmable environments to verify practical impact.

In summary, while the current formulation emphasizes analytical clarity and fundamental dynamics, we fully recognize the importance of evaluating finite-buffer effects in applied contexts and have planned corresponding theoretical and empirical developments.

**5. Why did you choose a truncated normal distribution for control-plane processing time? Was this based on real data, or was it used for simplicity?**

We appreciate the reviewer’s question regarding the selection of the truncated normal distribution for modeling control-plane processing time. This choice was guided by empirical observations derived from the real-world IoT telemetry dataset used in our study. Specifically, the control-plane delay samples exhibited right-skewed behavior with well-defined upper bounds – consistent with bounded execution time characteristics of programmable P4-based control logic, where timing determinism is enforced to ensure predictable latency.

Alternative heavy-tailed distributions, such as log-normal or Weibull, were initially considered, but they tended to overestimate the tail probability and lacked natural boundedness, which contradicted the observed behavior. The truncated normal distribution, in contrast, allowed us to capture the central moments (mean, variance), accommodate moderate asymmetry, and enforce a hard upper limit, thus closely reflecting the underlying system’s constraints.

Parameter fitting was performed using an expectation-maximization (EM) approach applied to empirical control-plane latency traces. The resulting distribution demonstrated a close fit to the empirical cumulative distribution function (ECDF), confirming its appropriateness. Therefore, the selection of the truncated normal was based not on convenience but on its empirical alignment with the control-plane processing characteristics observed in our data.

**6. Can your model handle other types of IoT traffic beyond the Kaggle dataset? Have you tested it on different scenarios?**

We thank the reviewer for this important question. While the Kaggle-based telemetry traces were used for initial calibration and validation due to their availability and representativeness of bursty and state-dependent traffic, the proposed model is designed to be flexible and generalizable to other classes of IoT traffic.

Specifically, the use of a BMAP for traffic modeling allows the framework to capture a wide range of arrival patterns, including:

- periodic sampling with random jitter,

- bursty telemetry exports (e.g., from INT or SNMP),

- multi-source aggregation flows,

- modulated or correlated arrivals typical of sensor-actuator feedback loops.

The phase-type and truncated normal service time models are also structurally versatile and can be recalibrated to reflect other execution environments, such as low-power edge nodes, event-driven microcontrollers, or gateway processing stages.

Although the current paper focuses on a representative 5G-IoT pipeline scenario, we have also conducted preliminary stress-testing of the model under synthetic traffic profiles with varying burst intensities, idle gaps, and cross-flow interference. These tests showed that the core model structure remained stable and that the delay trends followed expected theoretical behavior across scenarios.

Extending this work to fully support diverse real-world IoT workloads (including event-driven sensing, multimedia telemetry, or energy-constrained batch reports) is part of our ongoing research roadmap. In future work, we plan to incorporate trace-driven simulation with multiple datasets (e.g., from smart grids, industrial IoT, and smart home environments), and evaluate model robustness under varying scheduling and queuing policies.

**Responses to the comments of Reviewer 2**

We sincerely thank Reviewer 2 for the detailed and constructive feedback. Your insightful comments have significantly contributed to improving the clarity, rigour, and practical relevance of our manuscript. We greatly appreciate the balanced evaluation—acknowledging the strengths of the analytical approach—while also pointing out areas requiring further justification, empirical grounding, and clearer exposition.

**1. One key concern is the limited practical validation. While the use of real-world traffic data from the Kaggle repository is appreciated, the results are derived entirely from simulation and analytical modeling. There is no testbed evaluation, no experimental setup with actual P4 environments (even emulated), and no prototype or application scenario that demonstrates how the model would be used in practice. For a topic so closely tied to performance engineering, this disconnect between theory and application significantly weakens the impact of the results.**

We thank the reviewer for raising this important and valid concern. We fully agree that bridging the gap between analytical modelling and practical implementation is essential, particularly in the context of performance engineering for programmable, latency-sensitive 5G-IoT systems.

The core contribution of this paper lies in the development and empirical calibration of a modular analytical framework that captures key behavioural characteristics of hybrid programmable architectures. Specifically:

- Section 2 formulates a BMAP/PH/1-like queueing system with semi-Markov dual-path switching to reflect the split data/control plane processing in programmable P4 switches.

- Section 3 details the parameterisation of the model using real-world bursty telemetry traces from the Kaggle IoT repository. BMAP generator matrices were calibrated via moment matching and autocorrelation alignment, while service times were fitted to exponential, Erlang, and truncated normal distributions using MLE and EM-based methods.

- Figures 3–5 validate that the calibrated model reproduces empirical distributions and delay patterns.

- Figures 6–9 explore system dynamics under parameter variation (e.g., burstiness, switching rate, service shape), which aligns with theoretical expectations and confirms the tractability and sensitivity of the model.

We acknowledge, however, that this stage of work does not include a physical testbed or prototype. To address this gap and extend the impact of our results, we have initiated a two-track validation roadmap:

***1. Emulated experimentation***

We are building an emulated test environment using P4 bmv2 and Mininet, extended with INT (In-band Network Telemetry) support. The setup includes three virtual switches arranged in a dual-path leaf-spine topology with programmable match-action tables enabling path switching. Synthetic bursty traffic will be generated using Scapy and tcpreplay, simulating telemetry bursts of 20–50 packets per batch with variable inter-batch gaps.

Packet-level telemetry (including queue depth, hop delay, and path identifier) will be embedded via custom INT headers and exported through a gRPC-based P4Runtime interface to a dedicated telemetry collector. This will enable:

- Delay trend comparison across alternative paths;

- Validation of queue length variance and empirical-to-theoretical distributional alignment;

- Real-time detection of transient congestion episodes and queue saturation points, as predicted by the analytical model.

***2. Prototype validation on hardware platforms***

To support real-world validation of the model, we are preparing a programmable hardware testbed composed of:

- Intel Tofino 2-based programmable switch (e.g., Edgecore Wedge100BF-32X):

Enables deployment of P4 pipelines implementing dual-path (data/control) processing with externally-triggered control logic. The switch supports INT metadata export and per-stage queue monitoring, which facilitates validation of processing time distributions, queuing delays, and burst-response behaviours under realistic pipeline constraints.

- NetFPGA SUME board (Xilinx Virtex-7 690T FPGA):

Used to prototype finite-buffer queueing logic, emulate overflow conditions, and apply programmable burst patterns. The board allows hardware timestamping, stress-testing of queue thresholds, and microsecond-scale delay profiling – enabling model-to-hardware alignment under controlled conditions.

- Traffic generation and control host:

An x86 server equipped with Intel NICs, DPDK, Scapy, and Python-based control logic, used for generating diverse traffic patterns (e.g., correlated batches, telemetry-like bursts), injecting synthetic traces, and measuring response latency. The telemetry stack (based on P4Runtime, gRPC, and tshark) collects ECDFs, histograms, and queueing delay logs for cross-validation.

This platform will allow us to benchmark model predictions against real hardware behaviour and investigate buffer occupancy dynamics, switching interactions, and response under high-variance traffic conditions.

***3. Use-case driven evaluation***

In parallel, we are preparing applied scenarios where the proposed model will act as a predictive engine for adaptive control and monitoring in programmable 5G-IoT systems. These include:

- Edge-based telemetry aggregation in smart metering and environmental monitoring networks, where aggregated sensor bursts (e.g., from LoRaWAN gateways or Zigbee devices) are processed by P4-enabled edge nodes to detect processing bottlenecks and optimize batching intervals;

- Programmable congestion detection in access switches (e.g., in enterprise or smart city deployments), where model-derived delay estimates help dynamically tune ECN marking, queue thresholds, or flow redirection policies;

- Parameter sensitivity control in hybrid 5G-IoT gateways, such as load-aware path selection or control-plane offload decisions based on predicted delay growth under varying traffic burstiness, forwarding delays, or queue saturation levels.

These use cases are being prototyped on a virtual testbed and hardware emulator, and they will serve to evaluate how well the model generalises across heterogeneous device types, queuing policies, and traffic modalities.

We believe that this two-stage validation roadmap (covering both emulated and physical platforms) will meaningfully complement the current analytical contributions and broaden the impact of our work. While a detailed description of this hardware validation framework (including experimental architecture, traffic generation scripts, and telemetry feedback loops) would substantially expand the manuscript, we consider it beyond the scope of the present theoretical contribution. Therefore, we have concisely summarised the planned steps in the revised Discussion and Future Work section and will present the implementation and benchmarking results in a dedicated follow-up publication.

**2. Additionally, several assumptions in the model are overly idealized. For instance, the use of infinite buffer capacities and the assumption of no packet loss may simplify the derivations, but they also distance the analysis from real-world constraints in IoT networks. While the authors argue that such assumptions ensure analytical tractability, a discussion of their practical limitations (or a sensitivity analysis to approximate more constrained environments.**

We thank the reviewer for pointing out the limitations associated with some of the modelling assumptions, specifically, the use of infinite buffers and the exclusion of packet loss, in the current analytical framework. We agree that such assumptions represent idealised conditions that may diverge from certain real-world deployment scenarios in 5G-IoT networks.

These assumptions were introduced intentionally to prioritise analytical tractability and to enable closed-form evaluation of system dynamics in programmable switch pipelines. As discussed in our response to Reviewer 1 (Comment 2), the core objective of this study was to develop a mathematically rigorous, modular delay model tailored for hybrid data/control path architectures and bursty telemetry traffic (typical of programmable P4-based systems).

In particular, the assumption of infinite buffers reflects the internal per-stage buffer design of programmable hardware (e.g., Intel Tofino), where backpressure and head-of-line blocking are mitigated by hardware-level flow control and pipeline scheduling. While idealised, this abstraction allows us to analytically characterise queueing latency trends and sensitivity under controlled traffic bursts, as shown in Figs. 6–9.

Nevertheless, we fully concur that introducing more realistic buffer constraints would enhance the generalisability of the model. To that end, we have planned the following extensions as part of our future work:

- Theoretical generalisation: We will extend the current BMAP/PH/1 model to its finite-buffer counterparts, such as BMAP/PH/1/K or M^X^/G/1/K queues, where K reflects realistic buffer limits. These extensions will capture loss probabilities and occupancy distributions, enabling the study of overflow-driven behaviours.

- Sensitivity analysis: In future versions of the model, we aim to numerically examine how varying buffer sizes, scheduling policies (e.g., priority queuing, drop-tail), and admission control strategies affect delay and throughput under bursty traffic.

- Applied validation: These generalisations will be experimentally verified using our emulated and hardware-based platforms (described in detail in our response to Comment 1). In particular, the NetFPGA SUME board will allow emulation of queue overflow and loss conditions under programmable load injections, thereby validating the model’s extensions under constrained environments.

In summary, while the infinite-buffer assumption was a deliberate abstraction consistent with existing analytical models of programmable networks, we acknowledge its limitations and have defined concrete directions to address them in our theoretical and applied validation roadmap.

**3. From a statistical perspective, the modeling choices are mostly appropriate, but the justification for specific distributions, such as the truncated normal used to model control-plane processing delays, is insufficient. It is not clear whether this choice was based on data fitting, prior literature, or simply mathematical convenience. Moreover, the paper would benefit from including confidence intervals or a brief discussion of model sensitivity to parameter variations.**

We thank the reviewer for their thoughtful observation regarding the justification of distributional choices and the statistical robustness of the model. We agree that these aspects are essential for evaluating the reliability and generalisability of our framework.

First, regarding the use of the truncated normal distribution to model control-plane processing delays, this choice was empirically motivated by the characteristics observed in real telemetry traces. The delay distribution in the control path exhibited asymmetry with a clear upper bound – reflecting capped control response times or scheduling granularity in programmable pipelines. We therefore adopted the truncated normal distribution, as it balances analytical tractability with the ability to model bounded, skewed delay patterns. The parameters were fitted using the expectation-maximization (EM) method to align with the empirical cumulative distribution functions (ECDFs) of control-plane measurements. This approach has also been employed in related works on delay modelling for bounded-latency systems.

Second, in response to the reviewer’s request for more rigorous statistical analysis, we have substantially expanded Section 3 to include a dedicated sensitivity and uncertainty evaluation. This evaluation quantifies how the model responds to variation in key input parameters (such as batch size and service time) and provides statistical confidence bounds for the predicted QoS metrics:

- Table 1 reports the mean delay E[*T*] and mean queue length E[*Q*] under different traffic configurations, along with 95% confidence intervals obtained via bootstrap resampling (1000 iterations, 100 simulation runs per setting). These results confirm the statistical stability of the model's predictions and its internal consistency under realistic burst-processing conditions.

- Figure 12 presents a heatmap of expected delays under joint variation of batch size and service time. This two-dimensional analysis highlights nonlinear sensitivity patterns, revealing operational thresholds beyond which performance degrades sharply. These findings demonstrate the model's ability to capture compounding effects and serve as a practical tool for preemptive QoS tuning in programmable systems.

These enhancements directly address the reviewer’s concerns and show that our modelling choices were both empirically grounded and validated under variation. We believe this response strengthens the statistical foundation of the work and clarifies the rationale for the selected parameterisations.

**4. Regarding presentation, the manuscript is generally well-written in standard English, but the clarity of the exposition could be improved. Some sections (particularly the mathematical modeling) are quite dense and could be difficult to follow for non-specialists. Including a table of notation and a summary of key variables would help significantly. Additionally, restructuring some of the longer paragraphs and simplifying the transitions between mathematical and conceptual descriptions would improve the overall readability.**

We thank the reviewer for the constructive suggestions regarding the presentation and readability of the manuscript. In response:

- We have created a comprehensive nomenclature, which summarises the key variables and symbols used throughout the analytical sections. This section has been added after the Conclusions, to ensure easy reference without interrupting the main flow of the paper.

- We also performed a general round of language polishing and structural improvements, particularly in the mathematical modelling section. These include paragraph segmentation, improved transitions between conceptual and formal descriptions, and clarifying comments where appropriate.

- We acknowledge the importance of making the manuscript accessible to a broader audience and plan to continue refining the text in collaboration with the journal’s production team during the copyediting stage, once the paper is accepted for publication.

We hope these updates address the reviewer’s concerns and enhance the clarity and usability of the paper.
